# Catheter-Associated Candiduria: Aggregates, Microscopy, and CFU Variability

**DOI:** 10.1007/s11046-026-01060-x

**Published:** 2026-03-10

**Authors:** Stephan Steixner, Angelika Bauer, Rosa Bellmann-Weiler, Cornelia Lass-Flörl

**Affiliations:** 1 European Excellence Centre of Medical Mycology, Institute of Hygiene and Medical Microbiology, Medical University of Innsbruck, Innsbruck, Austria; 2 University Hospital of Internal Medicine II, Medical University of Innsbruck, Innsbruck, Austria

**Keywords:** Candiduria, Urinary tract catheter, Aggregates, Colony forming units, Diagnostic variability, Quality improvement

## Abstract

Diagnosis of catheter-associated candiduria is limited by absent colony-forming unit (CFU) thresholds and heterogeneous laboratory workflows, complicating patient management. Within a quality-improvement (QI) initiative we aimed to (1) implement and evaluate a standardised quantitative culture (QM) workflow for catheter urine, (2) compare QM with routine semi-quantitative culture (SQM) for CFU detection and reproducibility, and (3) assess whether visible urine aggregates predict *Candida* spp. presence. From February 2024 to February 2025, we analysed 222 yeast-positive urine samples from 74 catheterised patients. This pilot combined process standardisation, parallel SQM and QM testing, targeted microscopy of aggregate-containing samples, and species identification by CHROMID® *Candida* agar and MALDI-TOF. Agreement between methods, intra-patient CFU variability over three daily samples, and the predictive value of aggregates were assessed. Aggregates were present in 29/222 samples (13.1%), of which 13 (44.8%) contained *Candida* spp., mainly *C. albicans.* No reliable macroscopic features distinguished *Candida*-positive from -negative aggregates. QM and SQM showed poor agreement (Bowker *p* < 0.001, κ = 0.17). QM detected growth in samples reported as “sterile” by SQM and provided continuous CFU counts. Cohort-level median QM counts were stable, but 13 patients (17.6%) showed notable variability, with 5 (6.8%) fluctuating > 10^3^ CFU/mL. Process data suggested batching and variable pre-analytics as contributors to discordance. A standardised QM workflow revealed substantial discordance with SQM and highlighted pre-analytical variability. Visible aggregates are unreliable indicators for *Candida* spp. presence. Adoption of standardised quantitative culture, systematic microscopy, and structured lab-clinician communication may improve diagnostic consistency; prospective evaluation is warranted.

## Introduction

Urinary tract infections (UTIs) in catheterised patients represent a frequent and clinically important problem in hospitals, contributing to morbidity, antibiotic use, and healthcare costs [1–6]. Although bacteria are the dominant pathogens in most catheter-associated UTIs, *Candida* species are increasingly detected in urine cultures from hospitalised and immunocompromised patients and pose distinct diagnostic and management challenges [1, 7–9]. Unlike common bacterial pathogens, there is no universally accepted colony-forming unit (CFU) threshold for defining clinically relevant candiduria; suggested cut-offs vary widely and are inconsistently applied in practice [10–15]. This ambiguity complicates treatment decisions and can lead to both overtreatment and undertreatment.

Laboratory processes and pre-analytical handling further compound the problem. Semi-quantitative culture methods, batching of samples, variable vortexing and plating techniques, and the choice of media influence yeast recovery and reported CFU values, undermining comparability within and between institutions [16, 17]. Clinicians therefore receive microbiological reports that may not be reproducible or readily interpretable in the clinical context. Macroscopic features such as visible urine aggregates are commonly noted and poorly characterised, but have been associated with *Candida* spp. infections; their diagnostic value for *Candida* spp. infection has not been established and may be confounded by mixed cellular debris or bacterial co-growth [10, 18–20].

Quality improvement (QI) approaches that target laboratory workflows offer a pragmatic path to reduce diagnostic variability and to increase the clinical utility of microbiology reports. Standardising pre-analytical procedures and culture techniques can improve reproducibility, while parallel testing allows quantification of disagreement and identification of process steps that need correction. Embedding such changes within a QI framework enables to refine protocols and to evaluate impact on diagnostic consistency and clinical decision-making.

In this pilot prospective QI project performed at the Institute of Hygiene and Medical Microbiology, Medical University of Innsbruck, we sought to (i) implement and evaluate a standardised quantitative culture (QM) workflow for catheter urine, (ii) compare QM results with the routine semi-quantitative method (SQM) to quantify method agreement and identify process drivers of discordance, and (iii) characterise macroscopically visible aggregates to determine whether they predict *Candida* spp. presence. We analysed 222 yeast-positive urine samples from 74 catheterised patients collected over three consecutive days to assess intra-patient CFU variability and to generate actionable recommendations for laboratory standardisation and improved lab–clinician communication.

## Material and Methods

### Sample Collection

We prospectively analysed urinary-tract catheter (UTC)-associated urine samples collected between February 2024 and February 2025 from hospitalized patients at the University Hospital Innsbruck from whom urine cultures were requested by the treating clinicians due to suspected urinary tract infection. The indication for urine culture submission followed routine clinical practice and was not standardized or defined by predefined clinical criteria within this study. Samples were processed at the Institute of Hygiene and Medical Microbiology for routine UTI diagnostics. No systemic clinical data, including symptoms or indication details, were recorded as part of the study. From this pool, we included only yeast-positive cases that had three consecutive daily samples. A yeast-positive case was defined either by the presence of yeast-cells in native light microscopy, or a cultural prove. A subset of samples had macroscopically visible aggregates and were subjected to detailed microscopic and microbiological examination. Patients were excluded if they received antifungal therapy during the three-day collection period or if the catheter was changed or removed during sampling. The transport to the laboratory was performed immediately; aiming for ≤ 2 h between collection and processing. If a delay was unavoidable, samples were stored at 4 °C and processed within 24 h.

### Macroscopic and Microscopic Investigation of Urine Samples

**Fig. 1:**
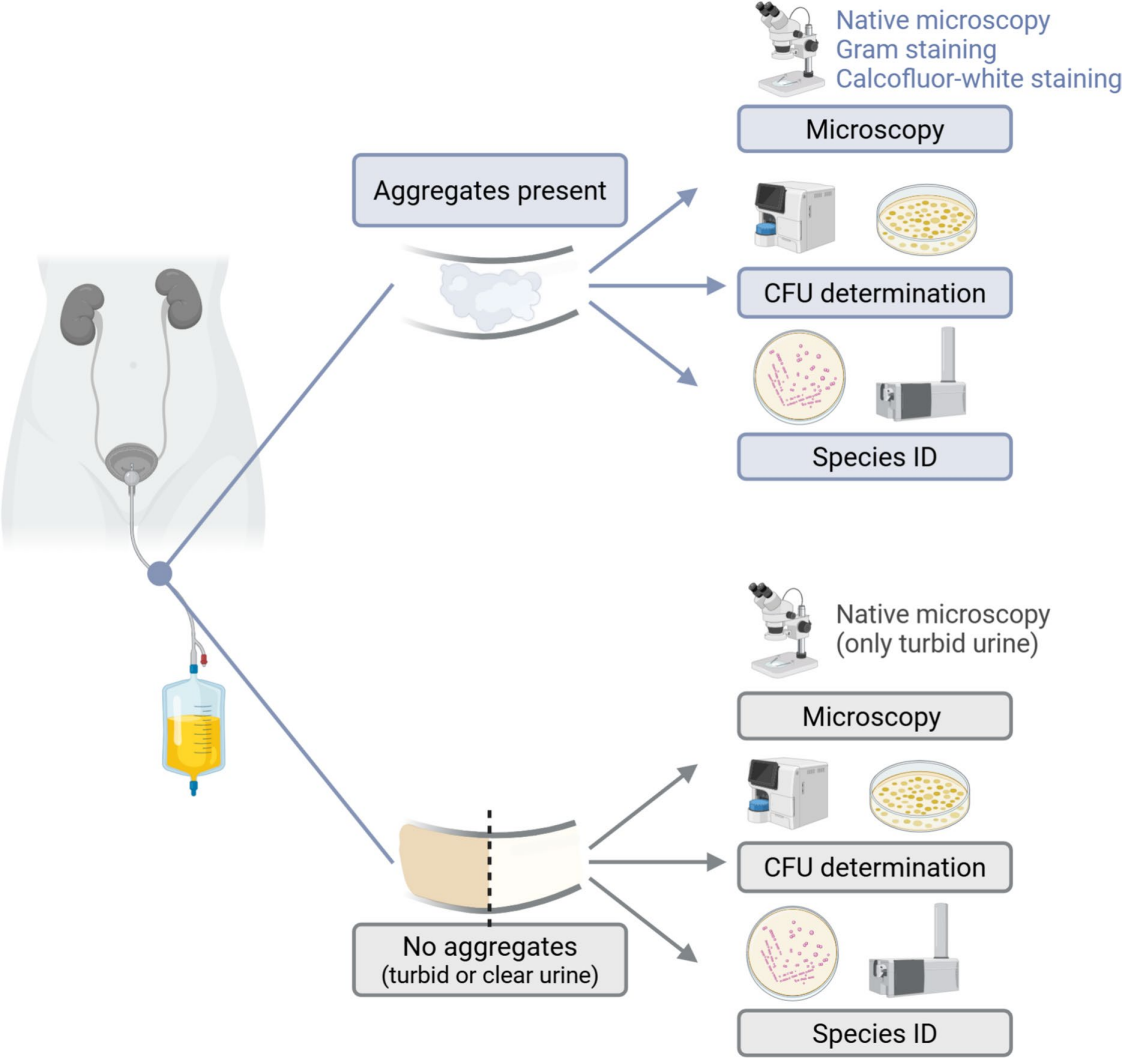
Workflow for the investigation of aggregates in urinary tract catheters. Urine samples were assessed macroscopically and classified as containing visible aggregates or not. Samples without aggregates were further classified as clear or turbid. All samples underwent: (1) microscopy (native light microscopy (except for clear urine samples); samples with aggregates additionally underwent Gram and calcofluor white staining), (2) colony-forming unit (CFU) determination, and (3) species identification on CHROMID® *Candida* agar and subsequent matrix-assisted laser desorption ionisation-time of flight (MALDI-TOF). Figure was created using BioRender [BioRender.com]

After macroscopic inspection, urine samples without visible aggregates were classified as clear (no turbidity or aggregates) or turbid. Turbid samples without visible aggregates were examined by native light microscopy; clear samples were not routinely examined microscopically. Samples with visible aggregates underwent detailed microscopy including Gram staining (PREVI® Color Gram system, bioMérieux) and calcofluor white staining (Fungi-Fluor® Kit, Polysciences Europe GmbH) to enhance detection of fungal elements (Fig. 1).

### Culture and CFU Counting from Urinary Samples

**Fig. 2:**
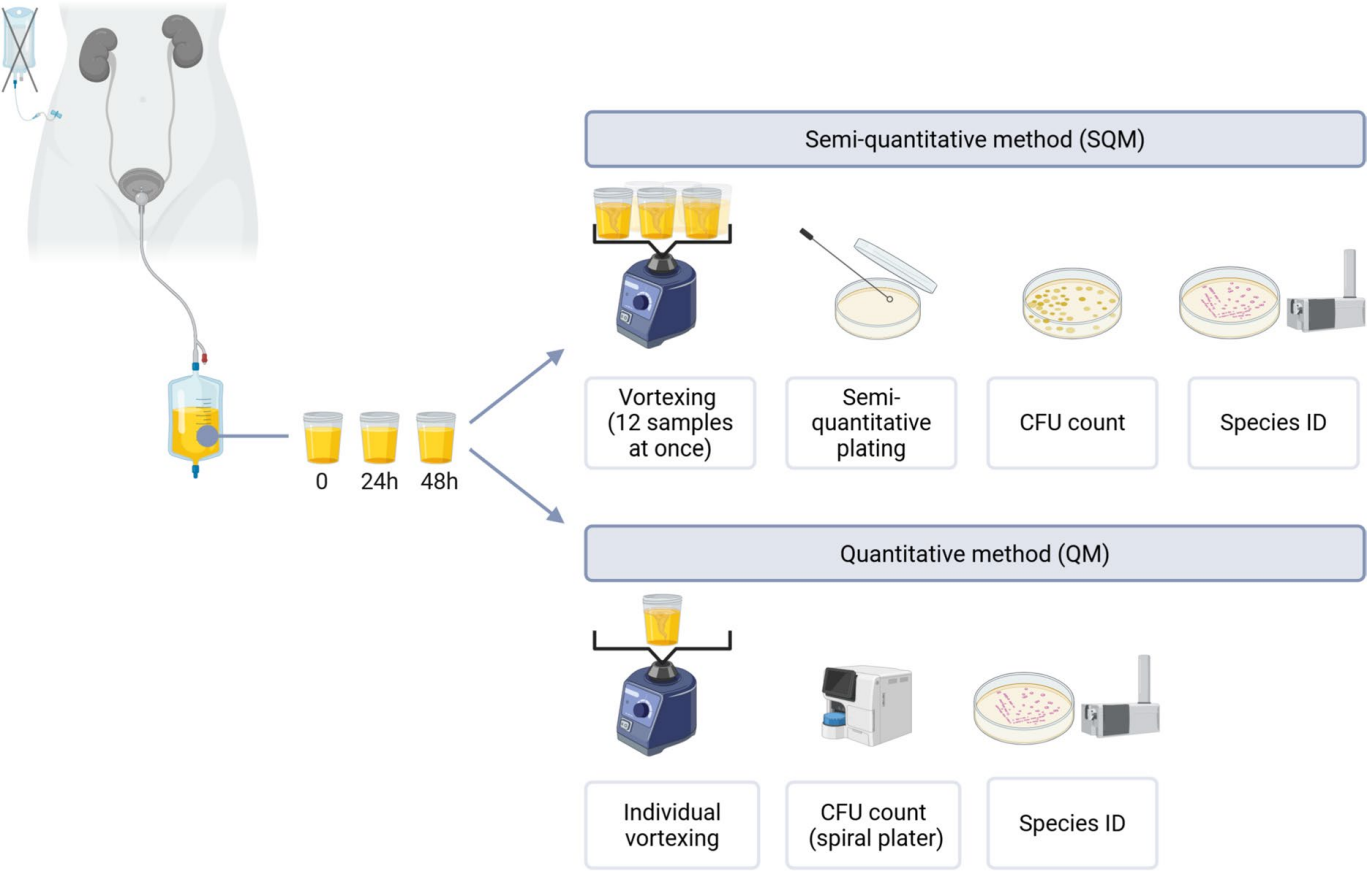
Comparative workflow of semi-quantitative (SQM) and quantitative (QM) colony-forming unit (CFU) determination. In SQM, 12 samples were vortexed together and plated in 10 µL aliquots onto Müller Hinton agar (grid). In QM, each sample was vortexed individually and plated on Sabouraud dextrose agar with gentamicin using a spiral plater; CFU/mL were calculated. Species identification was performed by CHROMID® *Candida* agar and matrix-assisted laser desorption ionisation-time of flight (MALDI-TOF). Figure was created using BioRender [BioRender.com]

Two approaches for urinary culture were applied as part of QI (Fig. 2). The SQM, which is used in routine urine analysis, was performed according to Schubert et al*.* [15]. Briefly, 12 samples were vortexed together in a batch; 10 µL aliquots were plated onto Müller Hinton agar in a defined grid pattern using a sterile loop for semi-quantitative distribution. The remaining plate surface was used to isolate colonies for identification. Plates were incubated at 37 °C for 48 h. After incubation, CFU were estimated and classified into four categories: “sterile”, “ < 10^4^ CFU/mL”, “10^4^–10^5^ CFU/mL”, and “ > 10^5^ CFU/mL”, defined by Schubert et al. [15]. The category of “ < 10^4^ CFU/mL” also included low-level growth of yeast.

Additionally, urine samples containing *Candida* spp. were plated on a CHROMID® *Candida* agar for colour-based differentiation of *Candida* species, followed by matrix-assisted laser desorption ionisation-time of flight (MALDI-TOF) identification (MALDI Biotyper smart system, Bruker Daltonik GmbH). Scores ≥ 2.0 were considered species-level identification.

For the quantitative method (QM), each sample was vortexed individually immediately before plating 50 µl on Sabouraud dextrose agar supplemented with 160 mg/L gentamicin using a spiral plater (WASP2, DonWhitley Scientific). Plates were incubated at 37 °C for up to 48 h and CFU/mL were calculated. Species identification was performed as above. Aggregates were also plated on Sabouraud dextrose agar containing gentamicin by spiral plating. If more than one yeast species was detected, CFU counts were performed on CHROMID® *Candida* agar.

### Statistical Analysis

Statistical analysis was performed using GraphPad PRISM version 10.4 where applicable. For method comparison, QM results were categorized into the same four SQM categories and Bowker’s test was applied; Cohen’s kappa was calculated and interpreted according to Landis and Koch [21]. Friedman’s test for matched measurements was used for CFU reproducibility across days. For CFU variability analysis we used only QM results. For the comparison of CFU counts between different *Candida* species and between visually turbid and clear urine samples, the Mann–Whitney U test was used, as the data were not normally distributed. A two-sided p-value ≤ 0.05 was considered statistically significant.

## Results

### Patient Characteristics

We included 222 yeast-positive UTC urine samples collected over three consecutive days from 74 patients. The cohort (Table 1) comprised 52 females (70.3%) and 22 males (29.7%) with a median age of 77.5 (28.0–98.0) years. Median time since last catheter change before the first sample was 7 (0–39) days. Fifty-five patients (75.3%) received antibiotic therapy; none received antifungal treatment.

**Table 1:** Patient characteristics

Patient characteristics	n = 74
Female (%): male (%)	52 (70.3): 22 (29.7)
Median age, (min–max) years	77.5 (28.0–98.0)
Median time since last catheter change until 1st sample (min–max), days	7 (0–39)
Antibiotic treatment, n (%)	55 (75.3)

### Macroscopic and Microscopic Investigation of Urine Samples

#### Macroscopic Examination of Urine Samples and Aggregates

**Fig. 3:**
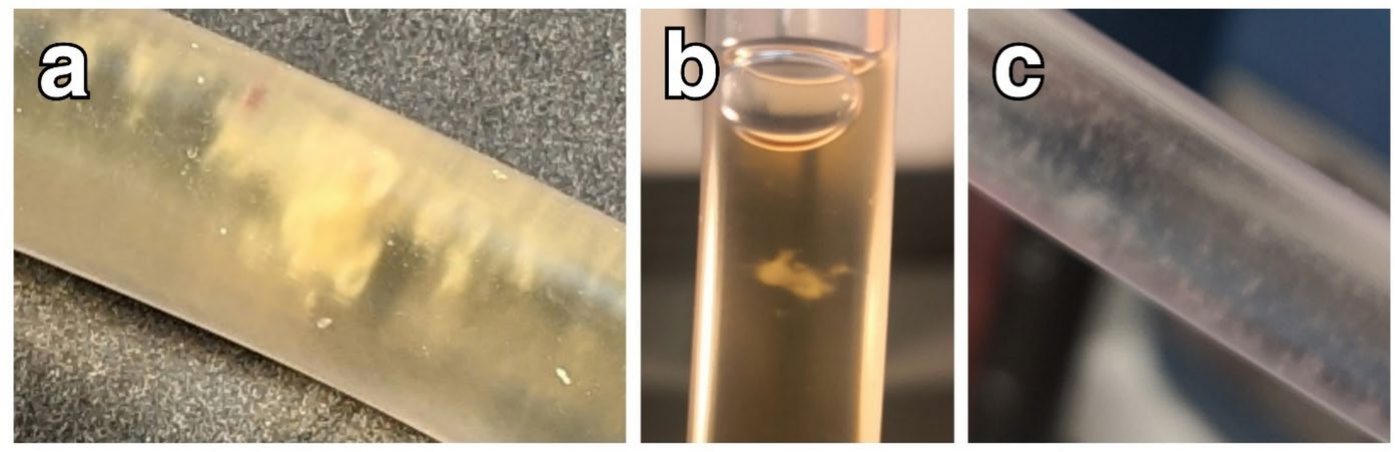
Different morphological appearance of white aggregates in urinary tract catheters. **a** Massive solid aggregates; **b** single solid aggregate; **c** small, fuzzy aggregates

Of 222 UTC urine samples, 29 (13.1%) contained visible aggregates and were examined microscopically with specific fungal stains. Aggregates were mostly white and appeared as massive solid clumps, single solid aggregates, or small fuzzy aggregates (Fig. 3).

#### Microscopic Examination of Urine Samples

**Fig. 4:**
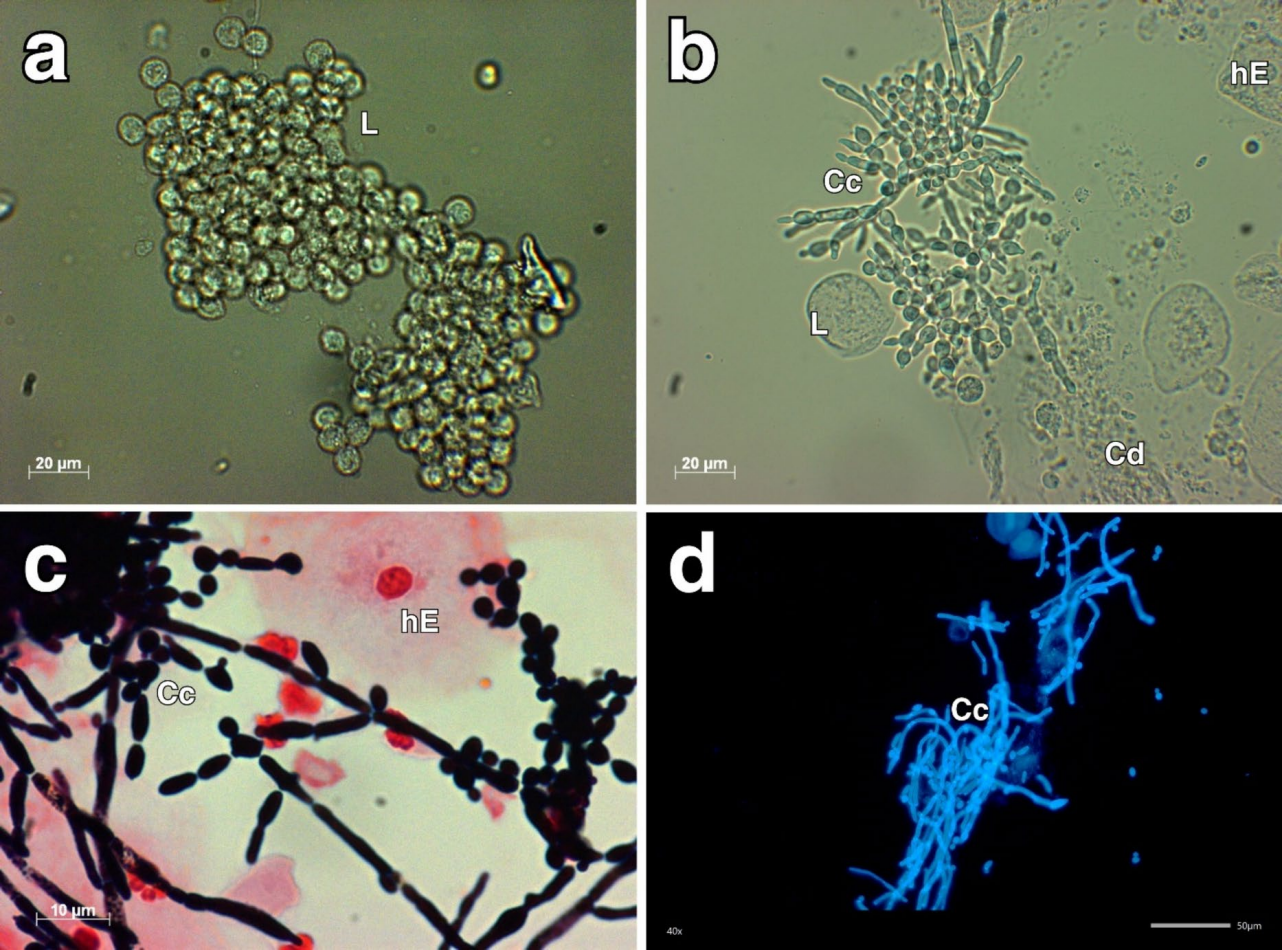
Microscopic imaging of white aggregates in urinary tract catheters. **a** Aggregate without *Candida* spp. cells; **b** aggregate with *Candida* spp. cells; **c** Gram staining of aggregate containing *Candida* spp.; **d** calcofluor white staining of aggregate containing *Candida* spp. L, leukocytes; Cc, *Candida* spp. cells; hE, human epithelial cell; Cd, cell debris

Forty-five (20.3%) samples were visually clear and not examined by native microscopy. Of the remaining 177 (79.7%) samples, 137 (77.4%) were positive for yeast in microscopy and 40 (22.6%) were negative for yeast in microscopy. In aggregate-containing samples (n = 29), aggregates consisted mainly of epithelial cells, leukocytes, and cell debris (Fig. 4). In 13 (44.8%) of these samples yeast cells were observed microscopically within the aggregates. No macroscopic features distinguished *Candida*-positive from *Candida*-negative aggregates. Representative Gram and calcofluor white stains are shown in Fig. 4.

### Microbiological Culture Results

**Fig. 5:**
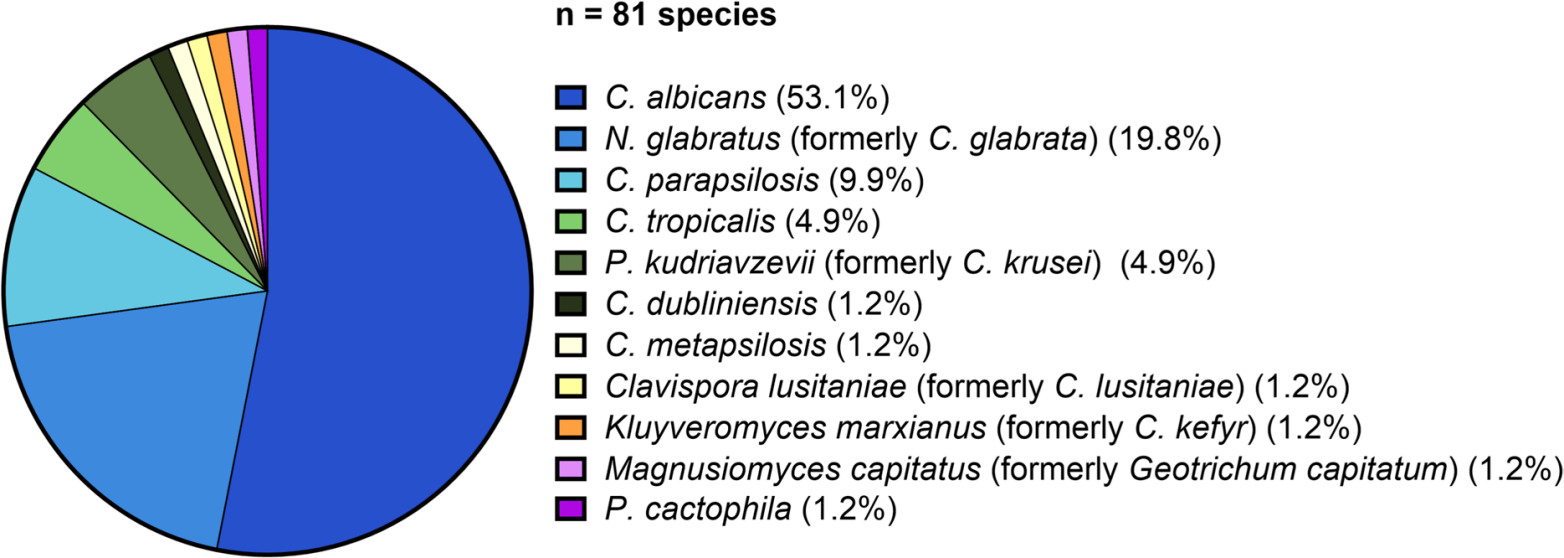
Fungal isolates (n = 81) from 74 patients with urinary tract catheters. Most prevalent species: *C. albicans* (n = 43, 53.1%), *N. glabratus* (n = 16, 19.8%), *C. parapsilosis* (n = 8, 9.9%), *C. tropicalis* (n = 4, 4.9%), *P. kudriavzevii* (n = 4, 4.9%). Others: *C. dubliniensis, C. metapsilosis, Clavispora lusitaniae, Kluyveromyces marxianus, Magnusiomyces capitatus*, and *P. cactophila* (each n = 1)

Across the 222 samples, 81 fungal isolates representing 11 species were identified (Fig. 5). Because intra-patient fungal composition did not change over the three consecutive days, results are summarized per patient. The most frequent species was *C. albicans* (n = 43, 53.1%), *Nakaseomyces glabratus* (n = 16, 19.8%, formerly *C. glabrata*), *C. parapsilosis* (n = 8, 9.9%), *C. tropicalis* (n = 4, 4.9%), and *Pichia kudriavzevii* (n = 4, 4.9%, formerly *C. krusei*), with several other species detected at very low frequency.

**Fig. 6:**
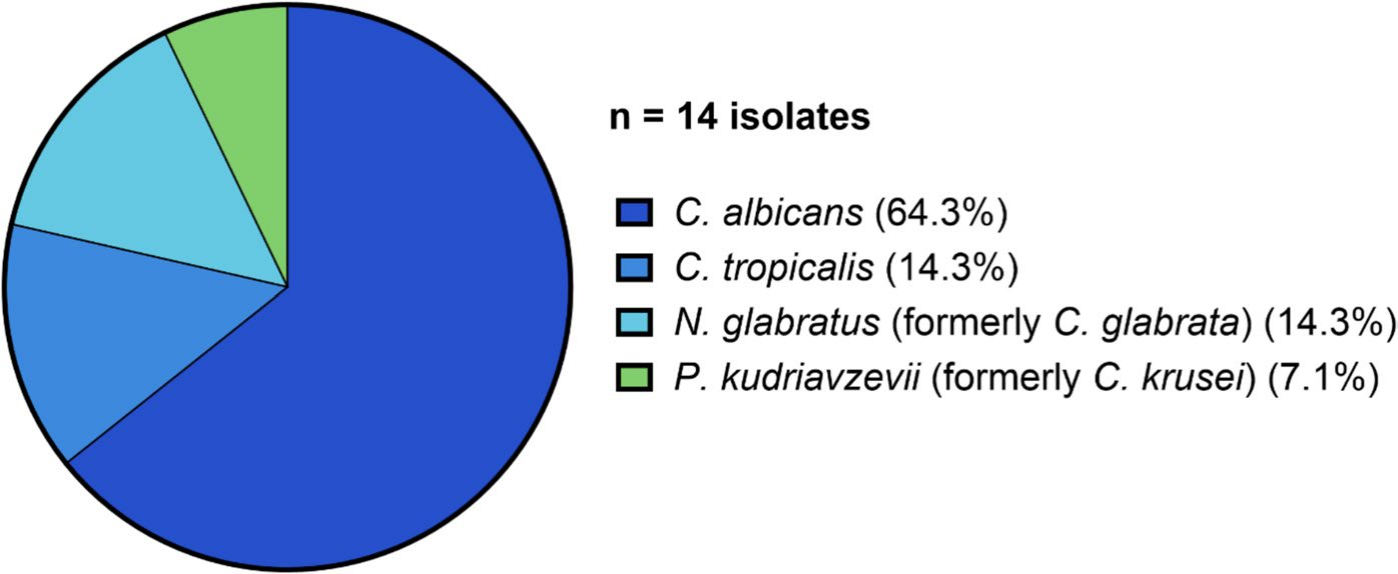
Detailed species identification of *Candida*-containing visible aggregates (n = 13) in urinary tract catheters. Most prevalent species: *C. albicans* (n = 9, 64.3%), followed by *C. tropicalis* (n = 2, 14.3%), *N. glabratus* (n = 2, 14.3%), and *P. kudriavzevii* (n = 1, 7.1%). One patient had two species (*C. albicans* and *N. glabratus*)

Among *Candida*-positive aggregates (n = 13), four *Candida* species were identified; one patient harboured two species (*C. albicans* and *N. glabratus*). *C. albicans* was most prevalent (n = 9, 64.3%), followed by *C. tropicalis* (n = 2, 14.3%), *N. glabratus* (n = 2, 14.3%) and *P. kudriavzevii* (n = 1, 7.1%) (Fig. 6).

### Analysis of CFU Counting

CFU comparison of visually clear and turbid urine samples revealed a significantly different counting (*p* < 0.001), with markedly higher CFU counts observed in turbid specimens. Further, patients receiving antibiotic therapy compared to those not receiving antibiotics, did not show a significant difference in CFU counts (*p* = 0.980).

#### Comparison of CFU Counts Obtained by Both Methods

**Fig. 7:**
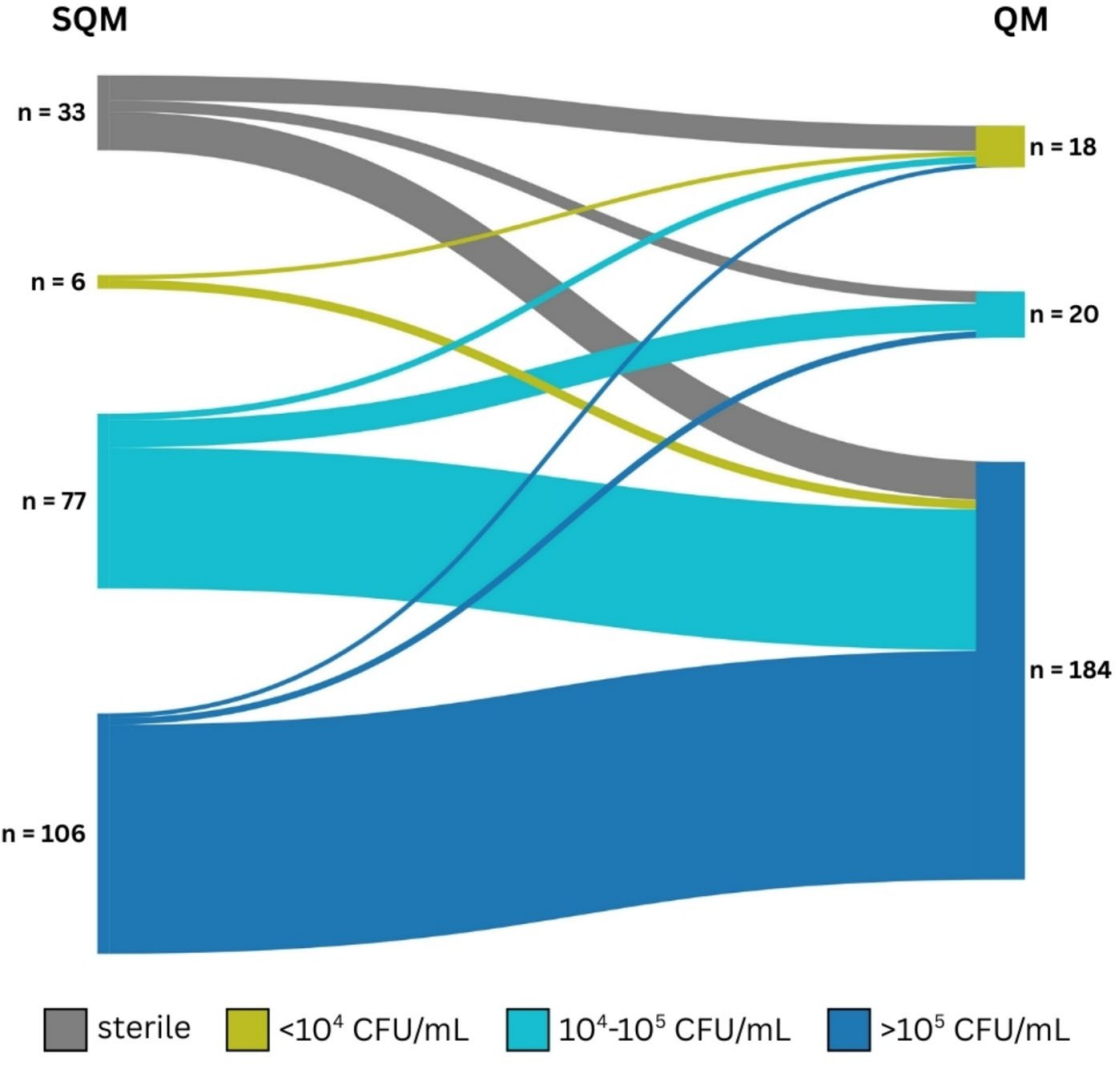
Sankey diagram comparing semi-quantitative (SQM; left) and quantitative (QM; right) colony-forming unit (CFU) categories for 222 urine samples. The diagram visualises transitions between categories and highlights shifts from “sterile” in SQM to high-count categories in QM. The diagram was created using SankeyMATIC [SankeyMATIC.com]

The SQM and QM comparison using a 4 × 4 contingency table showed significant asymmetry (Bowker’s test χ2 = 90.2, df = 6, *p* < 0.001), indicating systematic differences between the methods. Overall observed agreement was 52.5%, and Cohen’s kappa was low (κ = 0.17), indicating poor agreement beyond chance. The QM detected no samples as “sterile”, and 17 (7.7%) samples originally classified as “sterile” by SQM were classified as “ > 10^5^ CFU/mL” by QM. A Sankey diagram illustrates these reclassifications (Fig. 7). No significant difference in CFU counts was observed between *C. albicans* and non-albicans *Candida* isolates (*p* = 0.630).

**Fig. 8:**
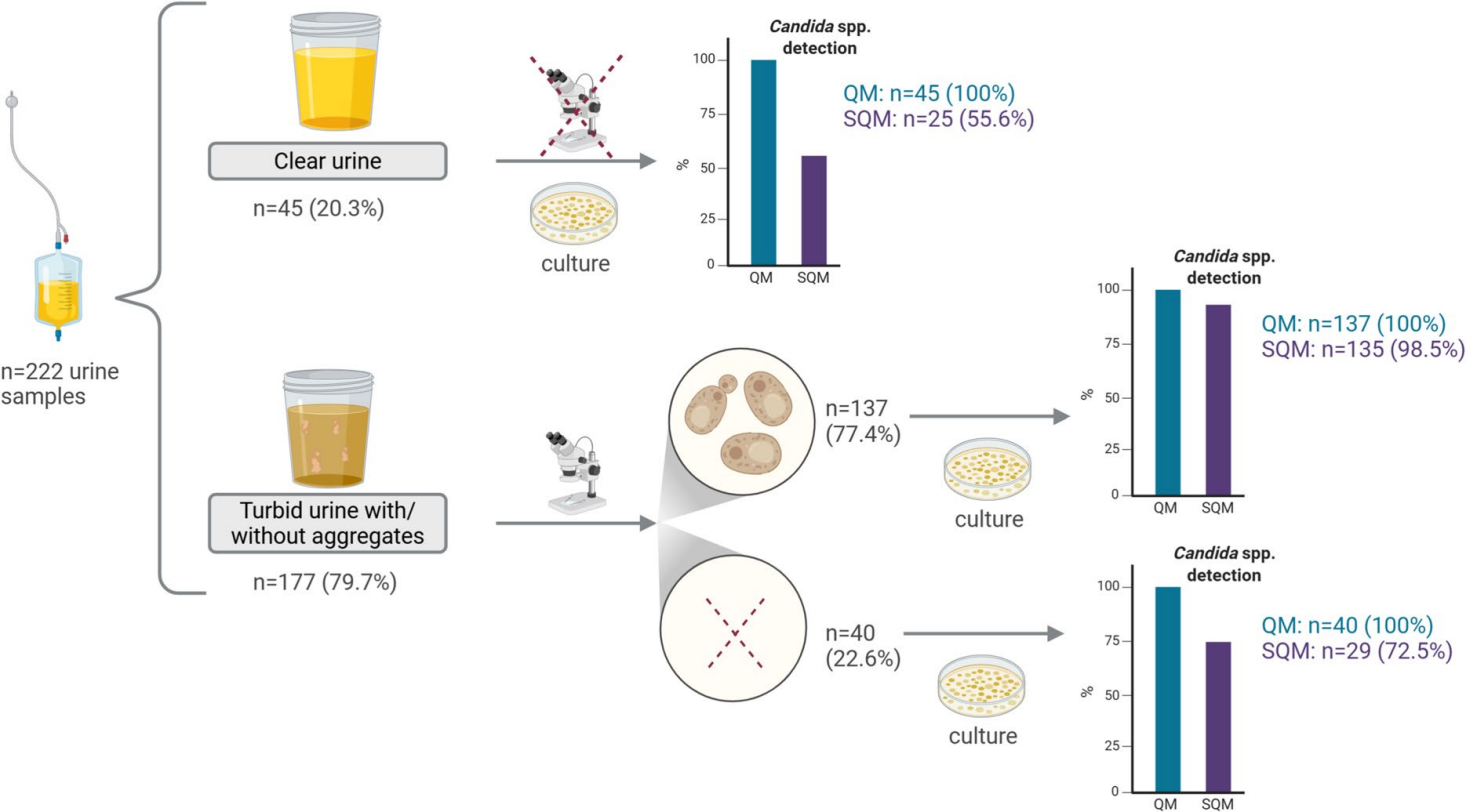
Schematic representation of the results of microscopy and semi-quantitative (SQM) and quantitative (QM; right) colony-forming unit (CFU) analyses of *Candida*–positive urine samples. Figure was created using BioRender [BioRender.com]

QM detected fungal growth in all urine samples (n = 222), including all visually turbid samples (n = 177, 79.7% of all 222 urine samples) and all visually clear urine samples (n = 45, 20.3% of all 222 urine samples) (Fig. 8). In contrast, SQM only detect fungal growth in 25 (55.6%) of the clear samples, all of which were positive by QM.

Among turbid urine samples (n = 177), native microscopy identified yeast cells in 137 (77.4%) samples, while 40 (22.6%) samples were microscopy-negative. Of the microscopy-positive turbid samples, 135 (98.5%) were culture-positive by both QM and SQM, whereas two (1.5%) samples remained sterile by SQM. Of the microscopy-negative turbid samples, fungal growth was detected in 29 (72.5%) of samples by SQM and in all samples by QM.

#### Variability of CFU Counts in Urine Samples Over a Three-Day Collection Period

**Fig. 9:**
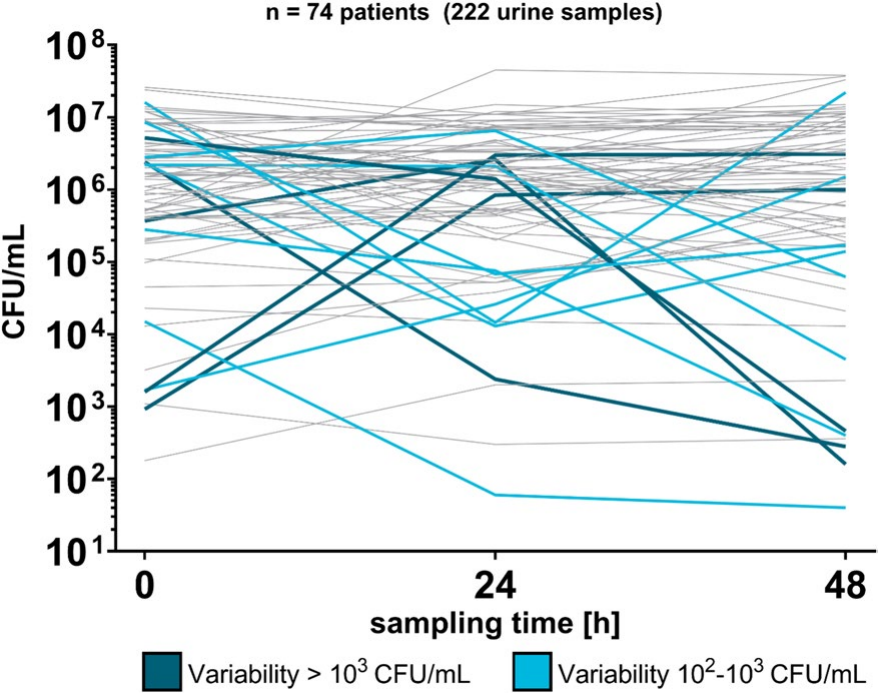
CFU/mL measured by quantitative method (QM) for three consecutive samples per patient. Grey lines indicate stable CFU across days (n = 61), light-blue indicates moderate variability (n = 8), and dark-blue indicates strong variability (n = 5)

Using QM data, cohort-level CFU counts were stable over three consecutive days (Friedman’s test *p* = 0.882). However, 13 (17.6%) patients displayed higher-than-expected intra-patient CFU variability. In detail, moderate variability (10^2^–10^3^ CFU/mL) was found in 8 (10.8%) patients and strong variability (> 10^3^ CFU/mL) in 5 (6.8%) patients (Fig. 9).

## Discussion

Framing this work as a laboratory quality-improvement project helps to focus interpretation on process drivers of diagnostic variability and on actionable steps to improve the clinical utility of urine mycology reports. In our pilot QI evaluation, three practical signals emerged: (1) macroscopically visible white aggregates are heterogeneous and are not a reliable standalone marker for *Candida* spp. in catheter urine; (2) semi-quantitative and quantitative culture workflows produced systematically different results, with QM detecting yeast in many samples labelled “sterile” by SQM; and (3) pre-analytical practices—notably batching and variable vortexing—are plausible contributors to discordance and intra-patient CFU variability.

Our microscopic and culture data demonstrate that nearly half of visible aggregates contain fungal elements, typically mixed with epithelial cells, leukocytes and host debris. Nevertheless, we observed no macroscopic features that discriminated *Candida*-positive from *Candida*-negative aggregates. From a QI standpoint, this reinforces that gross appearance alone should not trigger antifungal treatment or be used as a surrogate for infection; instead, visible aggregates should prompt standardised microscopic assessment and prioritised processing when clinically relevant. In line with this, species distribution among *Candida*-positive aggregates and in the overall cohort showed a predominance of *C. albicans*, consistent with published data [22–24]. In our study, the significant higher CFU counts observed in visually turbid compared to clear urine reflects a higher fungal burden in turbid samples. Although to our knowledge, no study clearly demonstrated this association between turbidity in urine samples with higher CFU counts of yeast cells, this association was consistently demonstrated for bacteria. For instance, Gadalla et al*.* showed that turbid (cloudy) urine was the strongest clinical sign for predicting a positive urine culture [25]. By using a more detailed scale to measure how cloudy the urine was, the accuracy of diagnosing bacterial urinary tract infections was improved and further linked to high counts of bacteria in urine cultures [25]. Importantly, in this study, clear urine was the strongest predictor for ruling out UTIs. This is supported by findings from Bulloch et al*.* [26], who found that clear urine on visual inspection yielded a negative predictive value of 97.3% for UTIs with bacteria. Therefore, diagnostic workflows should perform microscopy preferable of these turbid samples to faster diagnose the presence of pathogens in urine samples, as recommended by Schubert et al*.* [15].

The marked disagreement between SQM and QM has several practical implications for laboratory practice. QM offers continuous CFU/mL values, better recovery in selective media and—when combined with individual vortexing and rapid plating—greater sensitivity for low-level growth. A time delay between sample vortexing and plating may allow yeast cells to sediment, as yeasts have a relatively short sedimentation time and can settle quickly, affecting semi-quantitative counts [16].

By contrast, the routine SQM workflow used here involved batching and grid-plating that likely reduced fungal recovery and produced categorical results with limited resolution. For laboratories aiming to provide clinically useful quantitative fungal results, our data support adopting a standardised QM workflow or, at minimum, documenting and communicating the limitations of SQM-derived categories to clinicians.

Intra-patient CFU variability—observed in roughly 18% of patients in this pilot—merits attention as clinicians might be misguided by the assumption that a significant increase in CFU counts indicates a shift from colonization to active infection, leading to antifungal therapy being unnecessarily offered. Such a significant increase in CFU counts (> 10^3^ CFU/mL) was observed in 6.8% of the included patients, where the reasons are not yet fully understood. In patients with candiduria, particularly those with high CFU counts, additional diagnostic evaluations might be initiated [27].

Certainly, antimicrobial stewardship is dependent on the overall clinical status of the patient presenting with candiduria (including other underlying diseases as well as age). Individuals at higher risk for candiduria include neutropenic patients, older adults, women, patients with diabetes mellitus, those receiving antibiotic therapy, individuals with indwelling urinary catheters, and patients who have undergone prior surgical procedures [28, 29].

In critically ill patients, candiduria is often considered a potential precursor of disseminated candidiasis, regardless of clinical symptoms [28]. In those with indwelling bladder catheters, the presence of *Candida* spp. might reflect colonization rather than true infection and the removal of the bladder catheter is strongly recommended [28, 29].

From a process perspective, variability in CFU counts can arise from biological factors (intermittent biofilm shedding [30], mixed species dynamics [31]) and from pre-analytical and analytical variability (differences in vortexing, plating volume, media selection, and time-to-processing). Within a QI framework, these are addressable targets: standardise specimen handling (vortex time, plated volume, acceptable time-to-processing), prioritise rapid processing of samples with visible aggregates, and implement routine process controls and documentation to detect deviations.

This study has several limitations that should be considered. First, it is a single-centre, pilot investigation with a modest sample size and small event counts in key subgroups (e.g., *Candida*-positive aggregates, patients with large CFU fluctuations), which limits statistical power and generalisability. Second, clinical information linking microbiological findings to symptoms, antifungal treatment, catheter management, and patient outcomes was not systematically collected, preventing assessment of the clinical significance of *Candida* spp. detection or observed CFU variability. In addition, the indication for urine culture submission was clinician-driven and not standardized, as no predefined clinical criteria for suspected UTI were applied within the study. Consequently, urine samples may have been obtained for heterogeneous reasons, including nonspecific symptoms or routine evaluation in catheterized patients, further complicating interpretation of yeast growth in terms of infection versus colonization. Third, pre-analytical and methodological heterogeneity likely contributed to observed differences between SQM and QM: SQM used batch vortexing and grid-plating while QM used individual vortexing and spiral plating with selective media, and time delays between sampling and plating were not standardised. A further limitation is that differences in plated sample volumes (50 µl in QM versus 10 µl in SQM) affect the lower limit of CFU detection, as also demonstrated by others using 1 µl vs. 10 µl [32]. Furthermore, the effects of plating method and culture medium could not be analysed independently in this study. The comparison between SQM (Mueller–Hinton agar) and QM (Sabouraud dextrose agar with gentamicin) introduces a confounding variable, as differences in the CFU recovery rates of yeasts have been found dependent on the media composition [33–35], as well as the plating method, often showing higher detection rates of potential pathogens when an automated spiral plater is used compared to manual loop streaking methods [32, 36]. To investigate single effects of these different factors, further studies need to be performed. Another limitation is the feasibility of implementing a QM workflow instead of a SQM workflow in routine diagnostics in large laboratories. The QM workflow is more time-consuming than SQM and requires the use of a spiral plater. Fourth, “clear” urine samples were not routinely examined microscopically, introducing potential differential ascertainment bias. Taken together, these factors mean our findings should be regarded as hypothesis-generating; validation in larger, prospective, multicentre studies that standardise pre-analytical handling and include clinical endpoints and molecular/biofilm analyses is warranted.

## Conclusion

In this pilot cohort, macroscopically visible urine aggregates were not a reliable indicator of *Candida* spp. presence, and CFU results differed substantially between semi-quantitative and quantitative culture workflows. These results highlight that CFU interpretation is highly dependent on laboratory methodology and pre-analytical handling. Given the limited clinical data and pilot design, our findings should be considered exploratory; prospective studies that link standardised quantitative culture to patient symptoms and outcomes are needed before recommending specific CFU thresholds for clinical decision-making.

## Data Availability

All data supporting the findings of this study are available within the paper.
